# Regulatory T Cells Negatively Affect IL-2 Production of Effector T Cells through CD39/Adenosine Pathway in HIV Infection

**DOI:** 10.1371/journal.ppat.1003319

**Published:** 2013-04-25

**Authors:** Mohammad-Ali Jenabian, Nabila Seddiki, Ahmad Yatim, Matthieu Carriere, Anne Hulin, Mehwish Younas, Elnaz Ghadimi, Ayrin Kök, Jean-Pierre Routy, Alain Tremblay, Jean Sévigny, Jean-Daniel Lelievre, Yves Levy

**Affiliations:** 1 INSERM U955, Equipe 16, Créteil, France; 2 Faculté de Médecine, Université Paris Est Créteil, Créteil, France; 3 Vaccine Research Institute, Agence Nationale de Recherche sur le Sida et les hépatites virales (ANRS) HIV Vaccine Network (AHVN), Créteil, France; 4 Groupe Henri-Mondor Albert-Chènevière, Laboratory of Pharmacology and Toxicology, Créteil, France; 5 Faculty of Dentistry, McGill University, Montréal, Québec, Canada; 6 Division of Hematology and Chronic Viral Illness Service, McGill University Health Centre, Montreal, Québec, Canada; 7 Centre de Recherche en Rhumatologie et Immunologie, Centre de Recherche du CHU de Québec, and Département de Microbiologie-Infectiologie et d'Immunologie, Faculté de Médecine, Université Laval, Québec, Québec, Canada; 8 Groupe Henri-Mondor Albert-Chènevière, Immunologie Clinique, Créteil, France; National Institute of Allergy and Infectious Diseases, National Institutes of Health, United States of America

## Abstract

The mechanisms by which Regulatory T cells suppress IL-2 production of effector CD4+ T cells in pathological conditions are unclear. A subpopulation of human Treg expresses the ectoenzyme CD39, which in association with CD73 converts ATP/ADP/AMP to adenosine. We show here that Treg/CD39+ suppress IL-2 expression of activated CD4+ T-cells more efficiently than Treg/CD39−. This inhibition is due to the demethylation of an essential CpG site of the *il-2* gene promoter, which was reversed by an anti-CD39 mAb. By recapitulating the events downstream CD39/adenosine receptor (A2AR) axis, we show that A2AR agonist and soluble cAMP inhibit CpG site demethylation of the *il-2* gene promoter. A high frequency of Treg/CD39+ is associated with a low clinical outcome in HIV infection. We show here that CD4+ T-cells from HIV-1 infected individuals express high levels of A2AR and intracellular cAMP. Following *in vitro* stimulation, these cells exhibit a lower degree of demethylation of *il-2* gene promoter associated with a lower expression of IL-2, compared to healthy individuals. These results extend previous data on the role of Treg in HIV infection by filling the gap between expansion of Treg/CD39+ in HIV infection and the suppression of CD4+ T-cell function through inhibition of IL-2 production.

## Author Summary

Regulatory T cells (Treg) represent a subset of T lymphocytes and have a pivotal role in chronic viral infections and cancer by limiting immune activation. It has been shown that Treg are expanded in chronic HIV infected patients. However, the mechanisms of Treg immune-modulator functions are not clearly known. CD39 is an ectonucleotidase which converts the proinflammatory ATP signal into AMP and the immunosuppressive adenosine in concert with another ecto-enzyme CD73. We have previously reported that CD39/adenosine pathway is involved in AIDS progression. However, the mechanism of Treg immunosuppression through CD39 and its involvement in HIV pathogenesis remains unclear. We report here that Treg/CD39+ inhibits the production of IL-2, a cytokine that stimulates the growth of T lymphocytes, via CD39/Adenosine/cAMP enzymatic pathway. The signals induced by adenosine specific receptor A2AR, increase the intra cellular levels of cAMP. We show that cAMP inhibits CpG site demethylation of the *il-2* gene promoter. We found that T cells from HIV patients have a higher expression on A2AR as well as intra-cellular cAMP and a lesser capacity to produce IL-2 upon stimulation than healthy subjects. Our results contribute to elucidate the mechanisms by which Treg suppression occurs during HIV infection.

## Introduction

Regulatory T cells (Treg) play a dominant role in self-tolerance, control of autoimmune diseases and control of chronic infections by suppressing effector T cells activation, proliferation and functions [1]. Natural Treg derive from the thymus and are characterized by high levels of IL-2 receptor (CD25) and transcription factor FoxP3 and low levels of IL-7 receptor alpha (CD127) [2]–[5]. Induced Treg are heterogeneous and their phenotype and frequency vary across different disease states. They include interleukin-10 (IL-10) producing Tr1, transforming growth factor (TGF-β-expressing Th3 cells) [6], [7] and also Foxp3+CD39+ effector/memory Tregs [8].

The imbalance of T cell responses in favor of Treg can hamper efficient effector T cell responses as it has been observed in cancer and certain chronic infections [9]. In acute and chronic phases of HIV infection, a dual role for Treg has been reported due to their expansion [10]–[12]. Treg can suppress anti-HIV specific CD4+ and CD8 T cell responses by inhibiting cytokine production and cell proliferation [13], [14]. Increased Treg frequency at the mucosal site is accompanied by increased immune activation and decreased HIV-specific T-cell responses [15]. However, Treg can have a beneficial role by protecting HIV infected patients either at the primary or chronic phase of infection from the deleterious effects of HIV-induced chronic immune activation [11], [16], [17]. In HIV controllers, low frequencies of Treg have been associated with effective adaptive immune responses, but also with generalized immune activation and CD4 depletion [18].

Numerous mechanisms of Treg suppression have been reported [1]. These include secretion of inhibitory cytokines (IL-10, TGF-ß or IL-35), induction of apoptosis by IL-2 deprivation, perforin/Granzyme B or by CTLA-4 and GITR interactions pathways [1], [19]. Treg also use CD39 (nucleoside triphosphate diphosphorylase-1) and CD73 (ecto-5′-nucleotidase) for their suppressive activity. These ecto-enzymes hydrolyse extra-cellular pools of inflammatory ATP into adenosine diphosphate (ADP) and/or adenosine monophosphate (AMP) to adenosine [20]–[25]. Extracellular adenosine is known to be an important physiological regulator of the immune response [26], [27] by inhibiting T cell proliferation and IFN-γ/IL-2 production [28] and these effects are mediated through the adenosine-receptor A2A (A2AR) by stimulating the generation of intracellular cyclic AMP (cAMP) [28]. It has been recently shown that Treg inhibit HIV replication in conventional T cells through cAMP-dependent mechanisms [29]. We have recently evaluated the impact of CD39/adenosine pathway in HIV pathogenesis and reported that expanded Treg/CD39+ in infected patients correlate with immune activation and CD4+ cell depletion [30]. Importantly, we showed that these Treg exerted a strong suppressive effect on effector CD8 T cell functions and these inhibitory effects were relieved by using an anti-CD39 monoclonal antibody [30].

Here we explored the molecular mechanisms used by Treg/CD39+ cells to mediate their suppressive activity on CD4+ T cell function during HIV infection. Using co-culture experiments, we show that Treg/CD39+ cells inhibit IL-2 mRNA expression in activated effector CD4+ T cells. Importantly, this inhibition was partly reversed when CD39 enzymatic activity was blocked by an anti-CD39 mAb. We reasoned that this effect could be mediated through an epigenetic regulation of IL-2 expression involving cAMP-dependent mechanisms. We found that IL-2 inhibition mediated by Treg/CD39+ was correlated with a decrease in CpG demethylation of the *il-2* gene promoter. This effect was reproduced by using A2AR agonist as well as soluble cAMP. We also found that CD4+ T cells from HIV infected patients express high level of cytoplasmic cAMP and exhibit a lower frequency of demethylated CpG site of the *il-2* gene promoter following *in vitro* activation through the T cell receptor, when compared to healthy donors. Accordingly, A2AR expression was higher in *ex vivo* CD4+ T cells from HIV+ patients as compared to healthy controls. All together, these results make the link between Treg/CD39+ expansion and epigenetic mechanisms of IL-2 regulation.

## Results

### Treg/CD39+ inhibit IL-2 expression in activated T cells through inhibition of CpG site 1 demethylation of the *il-2* gene promoter

**Figure 1:**
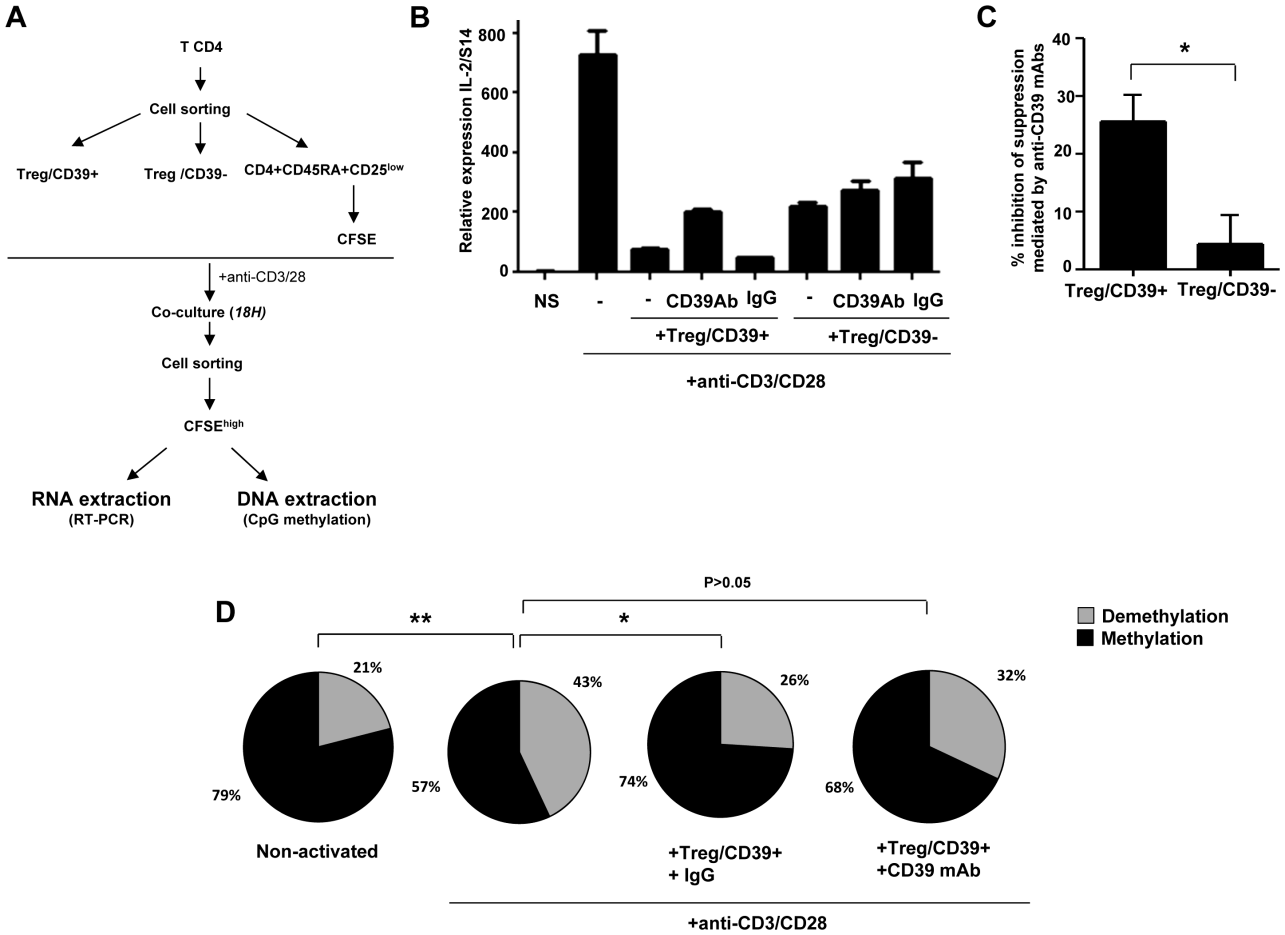
Treg inhibit IL-2 production via the CD39 pathway. (A) Using CD39 cell surface expression, Treg/CD39+ and Treg/CD39− were sorted and co-cultured with CFSE labeled CD4+CD45RA+CD25^low^ naive T cells (ratio ½). After overnight anti-CD3/CD28 (1 µg/ml) activation, CFSE naive T cells were resorted from co-culture and the impact of Treg on IL-2 production was evaluated by q-PCR. (B) A representative Figure of 3 independent experiments showing Treg/CD39+ suppression of IL-2 mRNA expression in anti-CD3/CD28 stimulated naive CD4+ cells. (C) Effect of anti-CD39 mAbs on suppressive function of Treg/CD39+ and Treg/CD39−. (D) The epigenetic changes on methylation of the unique specific CpG site of *il-2* gene promoter (n = 3) were studied by bisulphite modification of a mixture of DNA of anti-CD3/CD28 (1 µg/ml) activated naive T cells alone or co-cultured with Treg/CD39+ or Treg/CD39− in 3 independent experiments. Following molecular cloning and bulk sequencing, 20–30 colonies were analyzed for each experimental condition. *P<0.05, **P<0.01.

We first investigated whether Treg inhibited the expression of IL-2 in autologous anti-CD3 stimulated naive CD4+ T cells in co-culture experiments (Figure 1A). Naive CD4+ T cells were labeled with CFSE before co-culture with Treg populations and activated overnight with anti-CD3/28 mAbs. CD4+CFSE^high^ non-dividing cells were then FACS-sorted and IL-2 mRNAs were quantified by qRT-PCR. As shown in Figures 1B and 1C, Treg/CD39+ and Treg/CD39− inhibited dramatically mRNA IL-2 expression of anti-CD3/28 activated CD4+ T cells, but this effect was more pronounced, in the presence of Treg/CD39+ as compared to Treg/CD39−. Interestingly, in the presence of blocking anti-CD39 mAbs [29], [31], the suppressive function of Treg/CD39+ was decreased by 25±4% (P<0.05). As expected, these antibodies have no effect in co-cultures with Treg/CD39−. These results show that Treg/CD39+ inhibit, at least partially, the expression of IL-2 through the enzymatic activity of CD39. Moreover, we directly assessed CD39 enzymatic activity and measured ATP catalysis into ADP and AMP by HPLC and we have also quantified inorganic phosphate, which derives ATP hydrolysis. The results demonstrate the catalysis of exogenous ATP into ADP and AMP in the presence of purified Treg/CD39+ but not Treg/CD39−. Interestingly this catalysis was inhibited when an anti-CD39 mAb was added to the cells (Figure S1). We also evaluated the production of Adenosine via CD73 in our co-culture model. A significant increase in extracellular CD73 expression was observed in both naive CD4 T cells and Tregs upon overnight anti-CD3/28 mAbs stimulation (7±7.3 *vs.* 22.6±8.8% and 6±5.5 *vs.* 20.7±5.3, respectively, P<0.05, Figure S2). In line with these data, AMP was converted to adenosine in a specific manner as this conversion was totally inhibited by an inhibitor of CD73 enzymatic activity (6.3±6.4 *vs.* 0.13±0.3 µM, P<0.05; Figure S3).

Demethylation of the unique specific CpG site 1 in the *il-2* promoter is essential for inducing IL-2 production by TCR activation [32]. We therefore analyzed the methylation status of the CpG site 1 by the bisulfite genomic sequencing method on DNA extracted from anti-CD3/28 activated CD4+ T cells cultured or not with Treg/CD39+. Sequencing of *il-2* promoter gene region of 25–30 clones of each experimental condition was performed. As shown in Figure 1D, *in vitro* activation of CD4+CD45RA+CD25^low^ naive cells led to a higher frequency of demethylated CpG site 1 in the *il-2* gene promoter as compared to non-activated cells (43 *vs.* 21%; P = 0.01) whereas this effect was inhibited significantly when Treg/CD39+ were added to the co-cultures in the presence of an irrelevant IgG control (26%, P = 0.02). However, this inhibitory effect of Treg/CD39+ was partially reversed in the presence of an anti-CD39 mAb (32%, P>0.05). All together these data suggest that Treg/CD39+ suppress IL-2 expression in activated cells at the *il-2* gene promoter level.

### IL-2 expression in activated CD4+ cells can be modulated by A2AR agonists and antagonists

**Figure 2:**
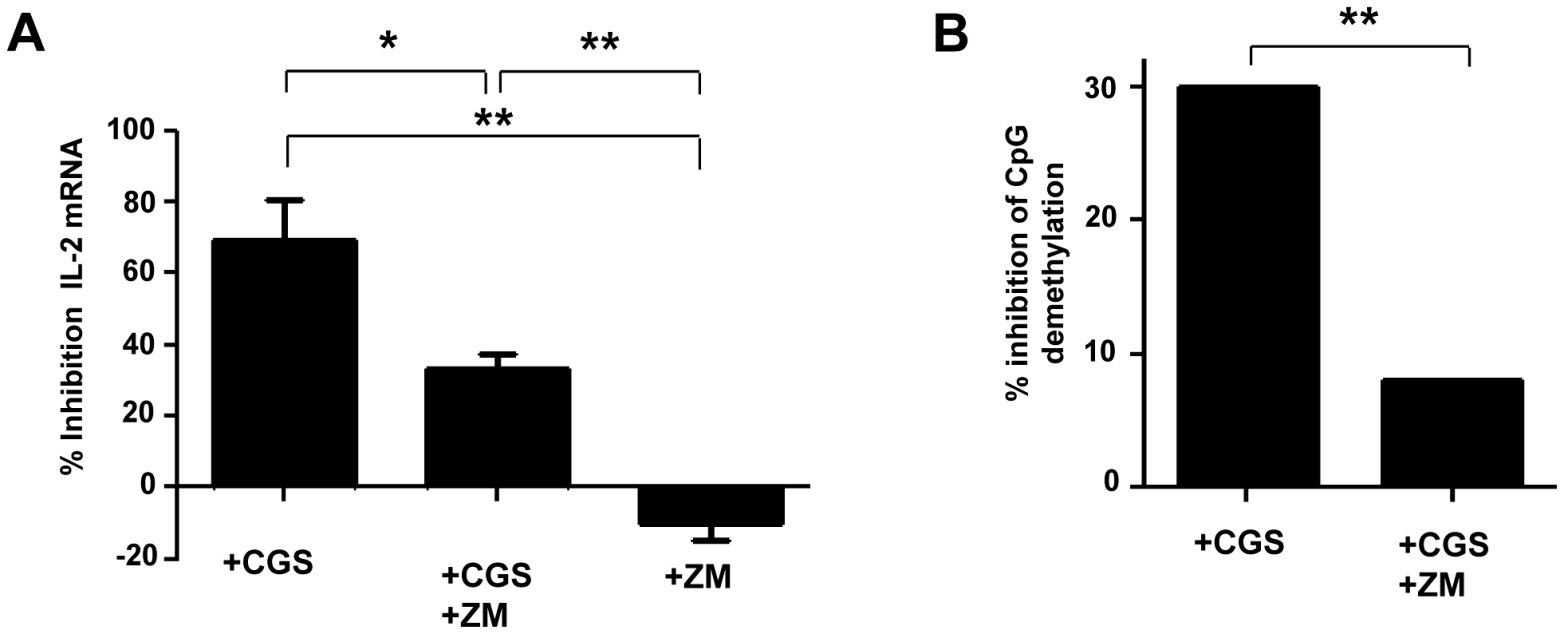
Inhibition of IL-2 production by A2AR induced signals during the activation of naive TCD4+ lymphocytes. CD4+CD25−CD45RA+ naive T cells were pre-incubated with A2AR agonist CGS 21680 (10 µM) and/or A2AR antagonist ZM 241385 (2 µM). After 6H of anti-CD3/CD28 (2 µg/ml) activation, IL-2 production was evaluated by q-PCR. (A) % of inhibition of IL-2 mRNA expression by A2AR stimulation (pooled data of 3 experiments). (B) Inhibition of demethylation of the unique specific CpG site of *il-2* gene promoter was performed by bisulphite modification of DNA following molecular cloning and bulk sequencing on anti-CD3/CD28 (2 µg/ml) activated naive CD4+ T cells pre-incubated with A2AR agonist CGS 21680 (10 µM) and/or A2A antagonist ZM 241385 (2 µM) in 3 independent experiments. 20–30 colonies were analyzed for each experimental condition. *P<0.05, **P<0.01.

To assess whether CD39/adenosine pathway is involved in the Treg/CD39+ mediated inhibition of IL-2 expression, we evaluated the effects of the A2AR agonist CGS21680 and A2AR antagonist ZM241385, on IL-2 expression of anti-CD3/CD28 stimulated-CD4+ T cells. We found that CGS inhibited significantly the expression of IL-2 (69±11.5% as compared to DMSO control condition). This effect was partially relieved when the A2AR antagonist ZM was added to cultures of activated CD4+ T cells in the presence of CGS (33±4%; P = 0.04 for comparison of CGS and CGS+ZM conditions) (Figure 2A). Of note, ZM alone did not alter the expression of IL-2 transcripts of activated CD4+ T cells.

Next we looked at the effects of A2AR agonist at the DNA level (Figure 2B). The frequency of demethylated CpG site 1 of the *il-2* promoter gene in the presence of anti-CD3/28 was 76% and became 53% in the presence of CGS which corresponded to 30% inhibition of CpG demethylation (Figure 2B). Addition of ZM before adding CGS to activated CD4+ T cells restored the frequency of demethylated CpG at the same level than activated CD4+ T cells (70% and 75%, respectively; P = NS). No effect of the DMSO as control of the vehicle of CGS and ZM was observed (percentage of demethylation around 75% in activated CD4+ T cells). These results show that the adenosine pathway is involved in the epigenetic regulation of the expression of the IL-2 gene in activated CD4+ T cells.

### Modulation of adenyl cyclase expression in stimulated naive CD4+ cells impacts on IL-2 expression

**Figure 3:**
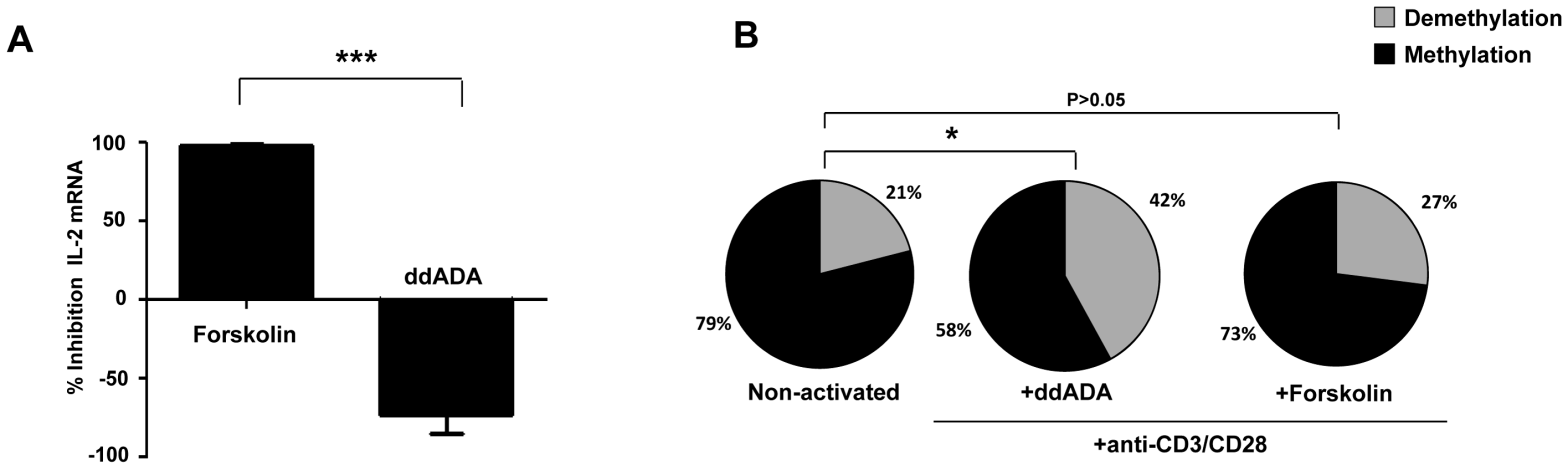
Activation of Adenyl cyclase inhibit IL-2 production by naive CD4+ T. CD4+CD25−CD45RA+ naive T cells were pre-incubated with the Adenyl cyclase inhibitor 2′,5′-DiDeoxyadenosine (ddADA) (200 µM), or the adenyl cyclase activator Forskolin (2 µM) for 30 mins. After 6H of anti-CD3/CD28 (2 µg/ml) activation, IL-2 production was evaluated by q-PCR. (A) % Inhibition of IL-2 mRNA expression by adenyl cyclase activator (pooled data of 3 experiments). (B) The epigenetic changes on methylation of the unique essential CpG site of *il-2* gene promoter. The cells were pre-incubated with ddADA (200 µM) or Forskolin (2 µM) for 6H before anti-CD3/CD28 mAbs activation in 3 independent experiments. 20–30 colonies were analyzed for each experimental condition. 20–30 colonies were analyzed for each experimental condition. *P<0.05, ***P = 0.0001.

In CD39/Adenosine enzymatic cascade, A2AR signaling activates intracellular adenyl cyclase enzyme, which increases intracellular levels of cAMP in conventional T cells [29]. To assess whether adenyl cyclase was involved in CD39-induced inhibition of IL-2 expression, we pre-incubated CD4+ naive T cells with adenyl cyclase inhibitor ddADA or adenyl cyclase activator forskolin, 30 minutes before anti-CD3/CD28 stimulation. As shown in Figure 3A, forskolin inhibited dramatically the expression of IL-2 transcripts in stimulated cells (97±2% inhibition). In contrast, inactivation of adenyl cyclase by ddADA favored IL-2 expression. Accordingly, we found that the frequency of CpG site demethylation of *il-2* gene promoter was 42% in ddADA conditions (P = 0.02 for comparison of ddADA and non-activated conditions) while it remains close to non-activated cells in the presence of forskolin (21% and 27%, respectively, P = 0.33; Figure 3B).

### cAMP inhibit T-cell proliferation and IL-2 expression at the promoter level

**Figure 4:**
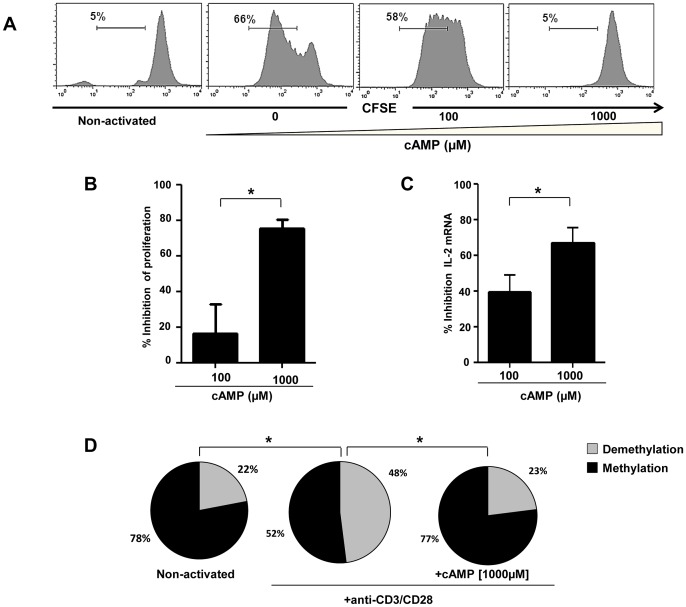
Inhibition of anti-CD3/CD28 mAbs stimulated naive CD4+ T cell proliferation and IL-2 production by cAMP. (A) Representative histograms showing the anti-CD3/CD28 stimulated proliferation of purified naive CD4+ T alone or treated with cAMP. Percentages of proliferating (CFSE^low^) CD4+ T cells are shown for each condition (one representative experiment out of 4 performed in triplicate); (B) Inhibition of CD4+ T cell proliferation by cAMP (pooled data of 4 experiments). (C) % Inhibition of IL-2 mRNA expression by cAMP (pooled data of 4 experiments). (D) The epigenetic changes on methylation of the essential CpG site of *il-2* gene promoter. The cells were pre-incubated with cAMP before anti-CD3/CD28 activation in 3 independent experiments. 20–30 colonies were analyzed for each experimental condition. *P<0.05.

To assess the impact of cAMP on CD4+ T cell proliferation, IL-2 mRNA expression and CpG site demethylation, CD4+ naive T cells were CFSE labeled and activated with anti-CD3/CD28 mAbs. As shown in a representative experiment performed in triplicate (Figure 4A), cAMP inhibited CD4+ T cell proliferation in a dose dependent manner (16±16% and 75±5% for 100 and 1000 µM respectively; Figure 4B). Similarly, cAMP inhibited IL-2 mRNA expression in activated CD4+ T cells a dose dependent manner (39±16% and 67±15% inhibition for 100 and 1000 µM respectively; Figure 4C). As shown in Figure 4D and as compared to non-activated CD4+ T cells, anti CD3/CD28 mAbs led to an increase of 26% in the percentages of demethylated CpG site 1 (P = 0.012), while no changes were observed when cAMP (1000 µM) was added to the culture (1% changes from non-stimulated conditions).

**Figure 5:**
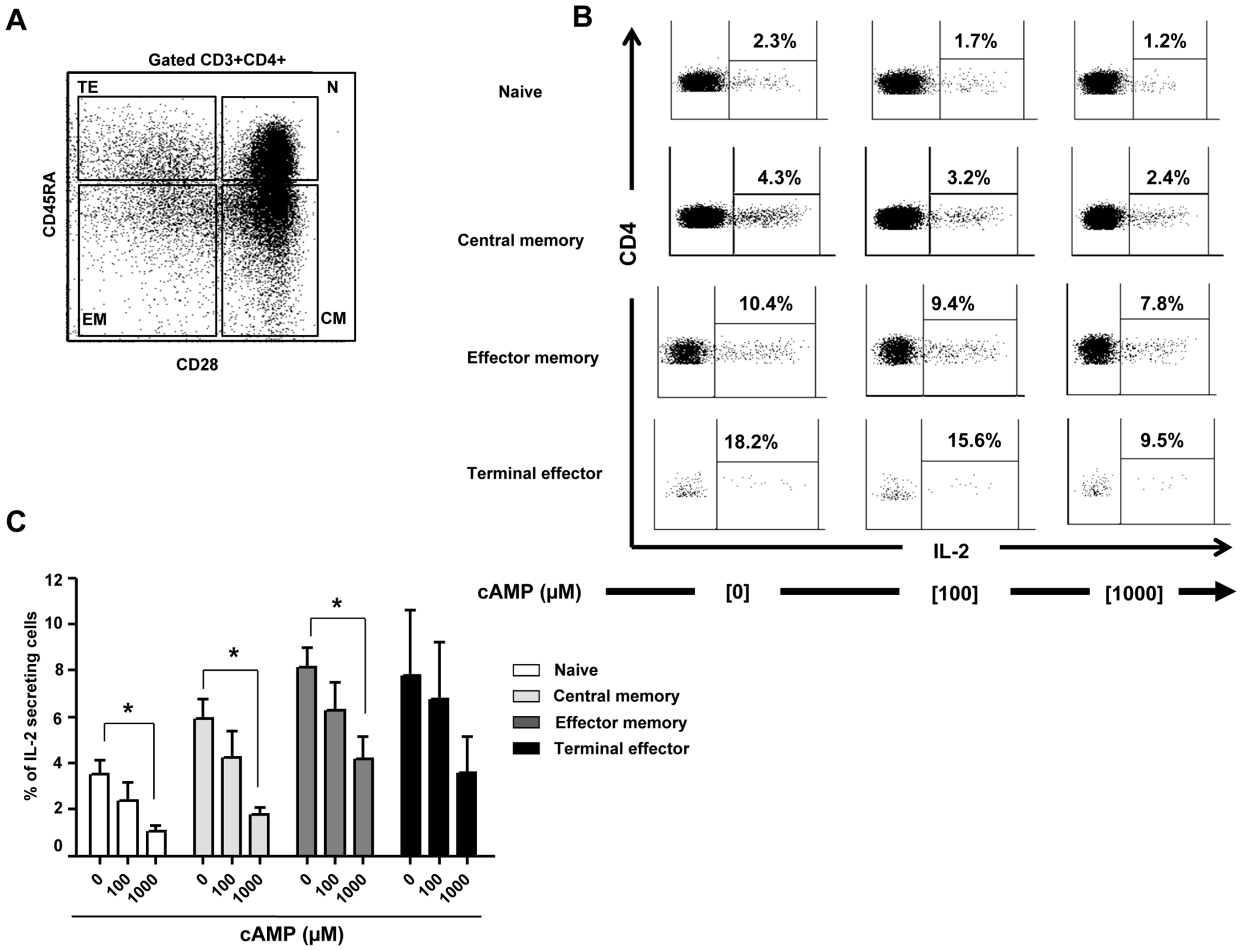
cAMP induced inhibition of IL-2 production by CD4+ T cell subsets. (A) Gating strategy. (B) Inhibition of intracellular IL-2 production by cAMP in all anti-CD3/CD28 (2 µg/ml) stimulated sub-populations of CD4+ T cells (representative figure of 5 experiments). (C) % Inhibition of intra-cellular IL-2 production (pooled data of 5 experiments) by all CD4+ T cells sub-populations. *P<0.05.

Next, we investigated whether the effects of cAMP on IL-2 mRNA expression translated to a decrease in the production of IL-2 by activated CD4+ T cells and if this effect was restricted only to naive CD4+ T cells. For this, naïve (N), central (CM), effector memory (EM) as well as terminally differentiated effectors (TE) (Figure 5A) were stimulated with anti-CD3/CD28 mAbs with or without different doses of cAMP. As shown in Figure 5B and C (for one representative experiment and pooled data, respectively), at the highest dose, cAMP inhibited by up to 75% the frequency of N and CM IL-2 producing cells as assessed by ICS assay (Figure 5C). This effect was also notable but less dramatic when cAMP was added to EM or TE (Figure 5C). Interestingly, the frequency of IL-2 producing cells following anti-CD3/CD28 stimulation within these latter subsets was higher than those of N and CM. Accordingly, *ex vivo* analysis of EM and TE FACS-sorted cells showed that the frequency of CpG site 1 demethylation reached 92–100% (data not shown).

### *In vitro* activated CD4+ cells from HIV+ patients were not able to demethylate *il-2* CpG site 1 and produce IL-2 due to their high levels of A2AR and intracellular cAMP

It is well known that in chronic HIV infection, T-cell dysfunction is characterized by reduced IL-2 production [33], [34]. The mechanisms leading to this defect remain unclear. Given our previous results showing that chronically infected HIV patients exhibit high levels of Treg/CD39+ [30], we investigated the potential implication of the CD39/A2AR/cAMP pathway in the regulation of IL-2 expression in CD4+ T cells purified from HIV-1 infected patients.

**Figure 6:**
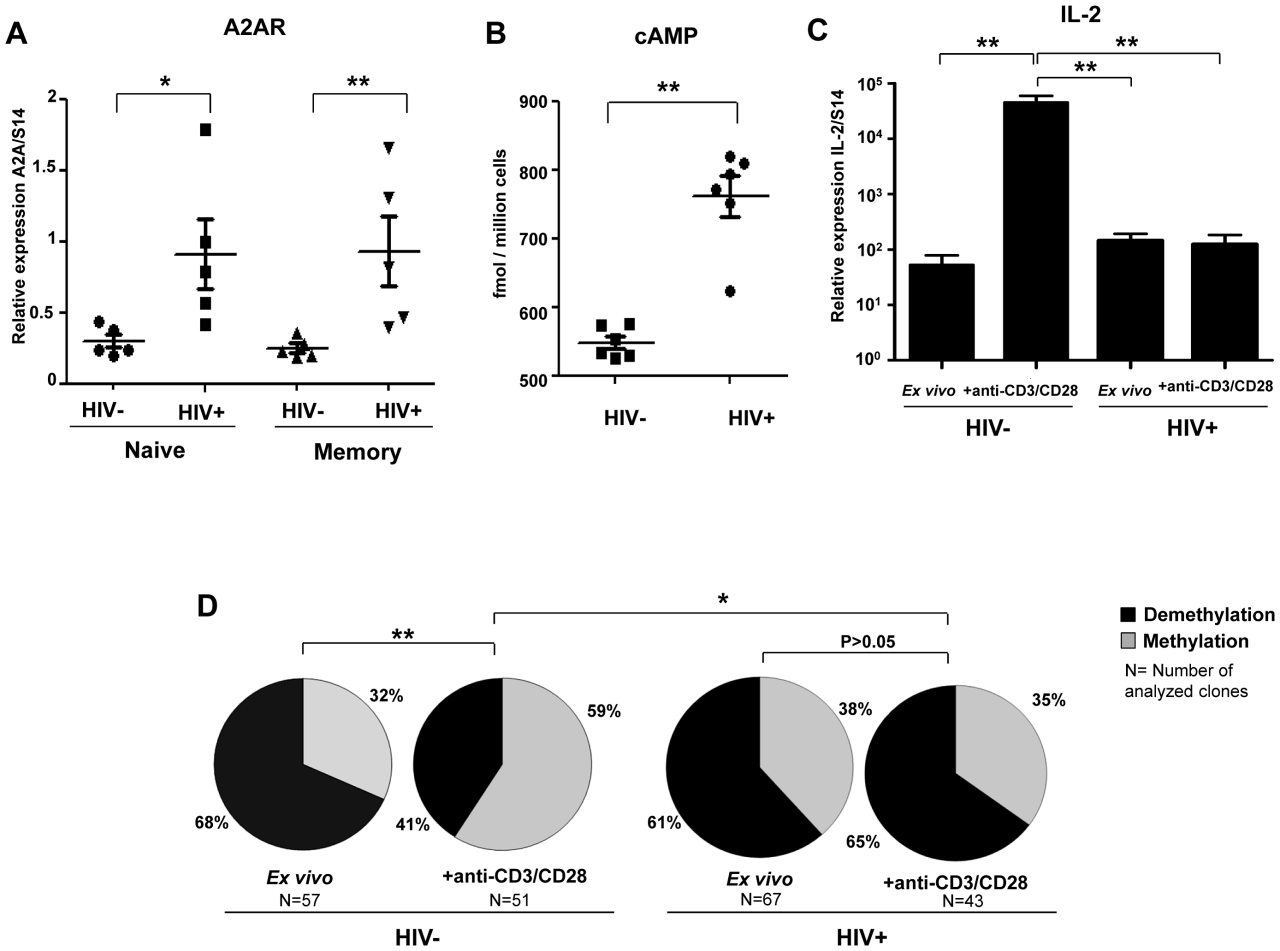
High levels of A2AR and endogenous cAMP in CD4+ T cells from HIV infected patients and lack of IL-2 production . (A) CD45RA+ and CD45RA− CD4+ T cells were purified from the blood of ART-naive HIV-infected patients (n = 5) and healthy controls (n = 5). A2AR mRNA expression was assessed using qPCR. Horizontal lines correspond to the mean for each data set. (B) CD4+ T cells were purified from the blood of ART-naive HIV-infected patients (n = 6) and healthy controls (n = 8). Intra-cellular cAMP is measured using the cAMP direct enzyme immunoassay from GE healthcare Biosciences. (C) Purified naive CD4+ T cells form HIV+ART- patients and healthy controls were stimulated with high doses of anti-CD3/CD28 mAbs (5 µg/ml) during 6H and IL-2 mRNA levels were quantified by RT-PCR. (D) The epigenetic changes on methylation of the unique essential CpG site of *il-2* gene promoter in naïve CD4+ T cells from HIV+ART- patients *vs.* healthy controls following anti-CD3/CD28 mAbs activation. To reach the technical limitation due to the low frequency of naïve CD4+ T cells in HIV+ART- patients, the Clonal analysis was performed on a pool of the extracted DNA from naïve CD4 T cells of HIV+ patients and healthy controls before and after CD3/CD28 mAbs activation. *P<0.05, **P<0.01.

Figure 6 shows that both naive and memory CD4+ T cells from HIV+ patients express significant higher levels of A2AR mRNA as compared to healthy controls (P<0.05 and P<0.01 respectively; Figure 6A). *Ex vivo* CD4+ T cells from HIV+ patients (n = 6) exhibit also higher levels of intra-cytoplasmic cAMP (mean 548.3±9.1 fmol/million cells) as compared to controls (n = 6; mean 761.6±29.4 fmol/million cells) (P = 0.002; Figure 6B). Therefore, we quantified IL-2 mRNA expression in purified naive CD4+ T cells from HIV+ART- patients (n = 6) and healthy controls (n = 8) following stimulation in the presence of high doses of anti-CD3/CD28 mAbs (5 µg/ml). As shown in Figure 6C, IL-2 mRNA levels were significantly lower in stimulated CD4+ T cells from HIV+ patients as compared to healthy controls (P = 0.004). In another set of experiments performed on a DNA pool obtained from *ex vivo* and anti-CD3/CD28 activated CD4+ T cells from HIV+ART- patients and healthy controls (n = 3 per group), the analysis of a large number of molecular clones (43 to 67 clones analyzed in each experimental condition) showed that the frequencies of demethylated CpG site 1 in non-stimulated cells were identical in HIV+ patients and healthy controls (P = 0.45, Figure 6D). Anti-CD3/CD28 activation increased significantly CpG demethylation in HIV− subjects (P = 0.006 for the comparison between *ex vivo* and activated conditions) but not in HIV+ patients (P = 0.67). Importantly, we showed that the status of patients (HIV− and HIV+) is significantly correlated with CpG demethylation of the il-2 promoter gene upon anti-CD3/CD28 activation (P = 0.02). All together these results demonstrate a constitutive high expression of A2AR and cAMP resulting in a clear inhibitory effect on CpG demethylation accompanied by the lack of IL-2 production in HIV+ART- upon anti-CD3/CD28 activation.

## Discussion

The mechanisms used for Treg's immunosuppressive function during the course of HIV infection are not completely elucidated. Recently we, and others, have shown an increased CD39 expression by Treg in HIV infected progressors compared to healthy controls [30], [35]. Moreover, we have shown that blocking CD39 enzymatic activity increased the production of cytokines by HIV-specific T cells. Genetic analysis of several cohorts of HIV-infected individuals showed a relative protection against the development of AIDS associated with CD39 genetic polymorphism [30]. All together these studies strongly suggest that the CD39/Adenosine pathway may play a detrimental role contributing to T cell dysfunction in HIV infection. Data presented here extend our previous results by demonstrating that Treg/CD39+ are potent suppressor of IL-2 production by effector T cells as compared to Treg/CD39−. By recapitulating the steps involved downstream of pericellular adenosine signals, we show that the involvement of the CD39/adenosine/cAMP pathway impacts on *il-2* gene promoter by inhibiting the demethylation of the unique specific CpG site 1 in *il-2* gene promoter, a seminal event for IL-2 expression in activated CD4+ T cells [32]. Furthermore we show that CD4+ T cells from HIV infected individuals are excessively sensitive to this pathway. Our data demonstrate that CD4+ T cells from HIV infected individuals are partially resistant to demethylation of CpG site 1 following an *in vitro* stimulation with anti-CD3/CD28 mAbs as compared to cells from healthy controls. Likely, this defect is associated with a lower expression of IL-2 by activated CD4+ T cells. Of note, as the majority of CpG site 1 in memory CD4+ T cells is already demethylated (more than 80%), in our *in vitro* anti-CD3/CD28 mAbs stimulation experiments, we used naïve CD4+ T cells to be able to study the induced modifications in CpG site.

Treg can induce cAMP in effector T cells by increasing adenosine levels in the microenvironment through CD39 and CD73 ectoenzyme pathways [8], [22]. In contrast to CD39 which is expressed on both human and murine Treg, CD73 is found only at the surface of murine Treg and this molecule is mostly absent on human Tregs membrane [8], [22], [36]. However, it is found in intra cellular compartment of human Tregs [36]. A rapid export of pre-formed CD73 to the surface of T cells due to the activation and its removal from the cell surface by an enzymatic cleavage has been reported [37]–[39]. Within human T cells, CD73 is mostly expressed by effector CD4 and CD8 cells [40] and might also be present in soluble form in the microenvironment [41]. It has been also shown an up regulation of CD73 mRNA in activated T cells [42]. In line with this observation, overnight co-culture experiments showed an increase CD73 expression at the surface of both naïve CD4+ T cells and Tregs upon anti-CD3/CD28 mAbs activation together with the conversion of both ATP and AMP into Adenosine. This molecule may be up regulated in inflammatory conditions and in cancerous tissues accompanied by high enzymatic activity [42]–[44].

Several studies have shown that adenosine plays an important non-redundant role in the regulation of T-cell activation via its specific A2AR [26], [45]–[47]. Signals induced by agonists of A2AR have an inhibitory effect on INF-γ and IL-2 production by effector T cells [28]. We confirm here our previous data [30] showing that both naive and memory CD4+ T cells from HIV-infected individuals express high levels of A2AR compared to healthy controls. Importantly, we show that by using an A2AR agonist, there was a specific and significant decrease in CpG site 1 demethylation of the *il-2* gene promoter followed by a decrease in IL-2 mRNA expression. These results help to make the link between pericellular adenosine signals through the purinergic receptor A2AR and dysfunction of Treg target cells in HIV infection.

Signals induced by A2AR agonists increase intracellular levels of cAMP [28] via activation of intracellular adenyl cyclase [48]. cAMP is known as an inhibitor of several cellular functions and immune responses such as T cell proliferation [49] and IL-2 production [50]. It has been shown in a murine AIDS model [51] and in *ex vivo* studies that T cells from HIV+ patients [52] exhibit higher levels of intracellular cAMP, resulting in a higher sensitivity of these cells to inhibition by cAMP analogues as compared to uninfected T cells [51], [52]. In accordance with this, we show significant increased intracellular cAMP levels in CD4+ T cells from HIV+ patients compared to healthy controls. Our data strongly suggest that the increased expression of cAMP in CD4+ T cells from HIV infected patients impairs IL-2 epigenetics regulation. First, we show that by using soluble cAMP with anti-CD3/28 stimulated CD4+ T cells we prevented both CpG site 1 demethylation of the *il-2* gene promoter as well as IL-2 mRNA and protein expressions. In line with our results, it has recently been shown in systemic lupus erythematosus, that cAMP has suppressive activity on IL-2 and IL-7 production through epigenetic modifications in IL-2 and IL-7 promoter genes [53], [54].

Increased levels of cAMP [28] are mediated by intracellular adenyl cyclase activity [48] in effector T cells [29], [55], [56]. cAMP has been described as a key component of Treg mediated suppression [57] and this suppression can be reversed by inhibition of adenylate cyclase activity [38]. In accordance with these studies, our results demonstrate that the adenyl cyclase activator forskolin, inhibits totally IL-2 mRNA expression and *il-2* specific CpG site 1 demethylation in activated T-cells. In contrast, adenyl cyclase inhibitor ddADA favors IL-2 production. These data suggest that Treg suppress IL-2 production through cAMP-dependent mechanism which directly impacts on CpG site 1 demethylation in the promoter regions.

The reason why CD4+ T cells from HIV infected individuals express higher levels of cAMP could be related to indirect or direct pathways. Likely, in the context of HIV infection several pathogenic pathways could favor the generation of adenosine and cAMP such as the generation of extracellular high ATP levels related to chronic activation and inflammation and the increased frequency of Treg/CD39+ in HIV [30]. Moreover, CD4+ T cells exhibit an increase in ATPase activity, a result that was associated with a higher percentage of cells expressing CD39+ [58]. Recently, in a model of acute SIV infection, a high expression of CD39 on CD8+FOXP3+CD25+ T cells was shown in the gut mucosa, a site of intense viral replication and inflammation [59]. On the other hand, Treg can also increase intracellular cAMP in effector T cells using an alternative mechanism, via gap junction, as it has recently been reported [29], [57]. These junctions allow intercellular communication between adjacent cells and the passage of ions and other molecules. It has been shown that resting T cells exhibit a low density of these channels. Whether chronically activated CD4+ T cells from HIV infected patients exhibit higher levels these channels warrants further studies.

From a physio-pathological standpoint, cAMP may play a dual role: a deleterious role by reducing HIV-specific antiviral immune responses [56] and T cell dysfunction as shown here and also a protective effect by limiting viral replication in infected cells and decreasing viral entry. It has been recently reported that increased cAMP levels through *in vitro* adenylate cyclase activation with forskolin diminished viral transcription and levels of HIV-p24Gag protein in activated T cells [60], [61]. Moreover, it is well known that during HIV infection there is an important decrease in CD4+ T cell proliferation and IL-2 production in viremic patients [34], but the mechanisms leading to this anergy remain unclear. Our data clearly show that even at high dose of anti-CD3/CD28 conditions, CD4+ T cells from chronically infected and untreated HIV patients, were not able to induce CpG site 1 demethylation of the *il-2* gene promoter which consequently impairs the production of IL-2. Further studies are needed to determine the role of CD39/adenosine/cAMP pathway in HIV acute infection but also in HIV infected patients under antiretroviral therapy, in order to evaluate whether these defects could be restored after treatment. It would be also interesting to evaluate whether CD39 mediated ATP hydrolysis as well as intra-cellular levels of cAMP differ according to the stage of HIV infection and disease progression notably in rapid progressors and elite controllers. Moreover, it will be also interesting to assess whether the different transcription factors necessary for an effective IL-2 expression, such as Oct-1 [32], which are recruited at the promoter level upon cell stimulation, are the same in HIV-infected patients with different clinical outcomes compared to healthy individuals. These studies will provide novel findings, which could help explain the transcriptional repression of the *il-2* gene in chronically infected HIV patients.

Altogether, our data strongly suggest that in viremic HIV+ patients, the decrease in T-cell proliferation and IL-2 expression is due in part to the inability of CpG site 1 to demethylate upon T cell stimulation. This defect is caused by increased intracellular cAMP, due in part to increased hydrolysis of inflammatory ATP by both expanded Treg/CD39+ and increased A2AR expression levels. Thus, our study establishes the link between Treg/CD39+ expansion and epigenetic mechanisms of IL-2 regulation in progressive HIV infection.

## Materials and Methods

### Patients and cell isolation

Blood samples from antiretroviral therapy (ART) naive HIV-infected patients and HIV-negative healthy donors were collected at The Clinical Immunology Department of Henri Mondor Hospital and the Regional Blood Transfusion Centre, Creteil, France. Ethical committee approval and written informed consent from all subjects were obtained before study initiation. Total CD4+ naive and memory T cells were purified using negative isolations kits from Miltenyi Biotec (Bergisch-Gladbach, Germany) according to the manufacturer's instructions. Treg/CD39+ and Treg/CD39− populations were FACS sorted using a moFlow cell sorter (Beckman-Coulter). The purity of sorted populations was >95%. Treg cells were defined by CD4+CD25^high^FoxP3+CD127^low^ T cells as we have previously reported [30].

### Co-culture of Treg and naive CD4+ T cells

Facs-sorted CD4+CD45RA+CD25− naive T cells were stained with 0.5 µM CFSE (Molecular probes, Eugene OR, US) and co-cultured with Facs-sorted Treg/CD39+ or Treg/CD39− at 1∶2 ratio, in the presence of 1 µg/mL anti-CD3 and anti-CD28 mAbs (Beckman Coulter, Villepinte, France). Total cell concentration was 3×10^5^/well (96-well plate) in a final volume of 200 µl. In some experimental conditions anti-CD39 mAb (10 µg/ml, clone A1, BioLegend, San Diego, LA) or IgG control was added to the cultures. After 18H, CFSE+ activated but non-divided CD4+CD45RA+CD25− T cells were Facs-sorted, then RNA and DNA extracted (Figure 1A).

### Measurement of CD39 ATPase activity

Treg/CD39+ or Treg/CD39− cells (5×10^4^ cells/well) were co-cultured with effector CD4+ T cells (10^5^ cells/well) in the presence or absence of anti-CD39 mAb or control IgG1 mAb (10 µg/mL) for 2 h. The cells were then washed with a phosphate-free reaction buffer (containing 0.5 mM CaCl2, 120 mM NaCl, 5 mM KCl, 60 mM glucose, and 50 mM Tris –HCl buffer, pH = 8) and ATPase activity was initiated by the addition of ATP or AMP at a concentration 100 µM in 200 µl of reaction buffer for 120 and 45 min respectively at 37°C. To block the internalization of Adenosine, the cells were pre-incubated for 15 min with the adenosine transporter inhibitor Dipyridamole (Sigma-Aldrich), at a concentration of 10 µM, prior to the addition of ATP or AMP. In some experiments a CD73 inhibitor, adenosine 5′-(α,β-methylene) diphosphate (Sigma-Aldrich), was added at a concentration of 100 µM 15 min prior to the addition of ATP or AMP. The released inorganic phosphate by hydrolysis of ATP was measured using the malachite green phosphate detection kit (R&D System, Minneapolis, USA) according to the manufacturer's instructions. In some experiments the supernatants were frozen (−80°C) until analysis by HPLC. This was done with either an Ultimate 3000 Thermofisher HPLC coupled with a UV detector on a reverse-phase column (Lichrospher 100-5 RP18 Macherey-Nagel) using a mobile phase gradient from 0 to 20% acetonitrile/50 mM KH2PO4 (pH = 6) containing 10 ml of Tetrabutylammonium phosphate 5 mM [62], or with a Beckman Coulter System Gold HPLC coupled with a UV system gold 168 detector on a reverse-phase column (Phenomenex Luna 3u C18(2) 100A,150 mm×4.6 mm) using a mobile phase composed of 25 mM TBA, 5 mM EDTA, 100 mM KH2PO4/K2HPO4, pH 7.0 and 2% methanol (v/v), at a flow rate of 1 ml/min [63].

### Proliferation assays

Different concentrations of cAMP (8-Bromoadenosine 3′,5′-cyclic monophosphate sodium salt, Sigma-Aldrich, Lyon, France) were pre-incubated for 30 min with CFSE labeled naïve CD4+CD45RA+CD25− T-cells. Cells were then cultured in 96-well plates and stimulated with 2 µg/ml of coated anti-CD3 and soluble anti-CD28 mAbs for 5 days. At day 2 of culture, cAMP was added in identical concentrations as day 0. The effect of cAMP on proliferation was evaluated measuring the percentage of CFSE^low^ dividing cells.

In some experiments, CD4+CD45RA+CD25− cells were pre-incubated with different reagents: 10 µM adenosine receptor agonist CGS 21680 (Sigma-Aldrich, Lyon, France) or 2 µM adenosine receptor antagonist ZM 241385 (Tocris bioscience, Bristol, UK) or with 200 µM of 2′,5′-DiDeoxyadenosine (ddADA) (Sigma-Aldrich) or 2 µM of Forskolin, an adenyl cyclase activator (Sigma-Aldrich) or DMSO for 30 minutes before activation with 2 µg/mL anti-CD3/CD28 mAbs.

### A2AR mRNA and IL-2 mRNA quantification

Total RNA was isolated from naive and total CD4+ T cells. qRT-PCR was performed using an ABI Prism 7500 Sequence Detection System (Applied Biosystems, Courtaboeuf, France) in 50 µL reaction with Platinum SYBR Green qPCR SuperMix-UDG w/ROX (Invitrogen) and 0.2 µM of each primer. S14 mRNA was used as a control to normalize each sample. Sequences of the IL-2-, A2AR- and S14-specific primers were forward: CGAGGGCTAAGGGCATCATTG, reverse: CTCCTTTGGCTGACCGCAGTT, forward: GGCAGACCGAGATGAATCCTCA, reverse: CAGGTCCAGGGGTCTTGGTCC and forward: GAATCCCAAACTCACCAGGA, reverse: TCAGTTCTGTGGCCTTCTTG respectively. The relative levels of IL-2 and A2AR mRNA were calculated using the 2^−ΔΔ*C*T^method.

### Intracellular cAMP quantification

CD4 T cells from HIV ART naive patients and healthy controls were isolated using negative isolations kits (Miltenyi Biotec). Intracellular cAMP levels were quantified in cell lysates (2×10^5^ cells/subject) using a commercially available assay (cAMP Direct Biotrak EIA, GE, Healthcare Biosciences, Pittsburgh, PA) according to the manufacturer's instructions.

### Flow cytometry

Anti-CD39-APC (clone TU66), -CD25-PE, -CD4-FITC, -CD3-Pacific Blue, -IL-2-PE-Cy7, -CD28-Percp-CY5.5 and -CD127-Biot/strepta-APCCy5.5 were from BD Biosciences (Le Pont de Claix, France). Anti-CD45RA-ECD was obtained from Beckman Coulter (Villepinte, France) and -FoxP3-Alexa 488 was obtained from ebiosciences (Montrouge, France). Cells were analysed on an LSR II (BD Immunocytometry systems).

### Clonal analysis of *il-2* methylated CpG-DNA

Total DNA was isolated from naive CD4+ T cells using DNeasy Kit DNA extraction kit (Qiagen, Duesseldorf, Germany). Genomic DNA was bisulfite converted using EpiTect Kit (Qiagen, Duesseldorf, Germany) according to the manufacturer's instructions. The unique essential CpG site (site 1) in the *il-2* gene promoter [32] was amplified by a PCR (forward GGAAAAATTGTTTTATATAGAAGG, reverse: TTCCTCTTCTAATAACTCTTTAA) followed by a nested-PCR (forward GGAAAAATTGTTTTATATAGAAGG, reverse: ATAAATATAAATAAAATCCCTCT). A clonal assay was performed for each experimental condition. Briefly, nested-PCR products were used for cloning into a pCR4-TOPO TA plasmid kit (Invitrogen, Carlsbad, CA, USA) and transfected in *Escherichia coli* according to the manufacturer's instructions. Colonies were grown on Luria-Bertani (LB) plates overnight at 37°C. Clones screening was done using PureLink Quick Plasmid Miniprep Kit (Invitrogen, Carlsbad, CA, USA) and plasmid DNA was prepared for sequencing analysis. Sequence analyses were done with SeqScape software (Applied Biosystems, Foster City, CA, USA) on at least 25–30 clones from each sample.

### Statistical analysis

The non-parametric Mann-Whitney *U*, Fisher's exact and paired T tests were used for statistical analyses (GraphPad Prism 5.0 statistical software). A P-value <0.05 was considered as significant.

## Supporting Information

Figure S1 **Differential ATPase activity of Treg/CD39+ or Treg/CD39−.** FACS-sorted Treg/CD39+ or Treg/CD39− cells were co-cultured with effector CD4+ T cells in the presence or absence of anti-CD39 mAb or control IgG1 (10 µg/mL) for 2 h. The cells were then washed with a phosphate-free reaction buffer and ATPase activity was initiated by the addition of ATP at a concentration 100 µM in 200 µl of the phosphate free reaction buffer for 15 min at 37°C. The released inorganic phosphate by hydrolysis of ATP was measured using the malachite green phosphate detection kit (R&D System, Minneapolis, USA) according to the manufacturer's instructions. Histograms represent Treg/CD39+ capacity to hydrolyse ATP comparing to Treg/CD39−. CD39 mAb inhibits the ATPase activity of CD39 in a specific manner (pooled data of 4 independent experiments, * P<0.05).
(TIF)

Figure S2 **Increase of CD73 expression following overnight anti-CD3/28 mAbs stimulation.** FACS-sorted naive CD4+ T cells and Treg/CD39+ cells were activated separately by anti-CD3 and anti-CD28 mAbs (1 µg/mL). After 18H of activation, the cells were washed and stained by anti-CD73 mAb. (A) A representative figure of 5 independent experiments showing an increase of CD73 expression upon anti-CD3/CD28 mAbs stimulation. (B) Histograms represent the increase of CD73 expression upon overnight anti-CD3/CD28 mAbs stimulation. (pooled data of 5 independent experiments * P<0.05).
(TIF)

Figure S3 **Hydrolysis of ATP or AMP into Adenosine in a co-culture of Treg/CD39+ and naïve CD4 T cells.** FACS-sorted Treg/CD39+ or Treg/CD39− cells were co-cultured with anti-CD3/28 mAbs stimulated naïve CD4+ T cells in the presence of 10 µM Dipyridamole to block the transport of Adenosine inside T cells prior to addition of 100 µM ATP or AMP, in 200 µl of RPMI. The cells were incubated for 120 min. with ATP or 45 min with AMP at 37°C, then the hydrolysis of exogenous ATP measured by HPLC. (A) A representative HPLC profile of 4 independent experiments (using a Beckman Coulter System Gold HPLC and a Phenomenex Luna 3u C18 (2) 100A, 150 mm×4.6 mm column) showing the ability of Treg/CD39+ to convert ATP into adenosine (Top panel). Addition of the inhibitor of CD73 enzymatic activity (adenosine 5′-(α, β-methylene diphosphate) inhibits the production of Adenosine in a specific manner (Middle panel). No hydrolysis of exogenous AMP into Adenosine was observed when Treg/CD39− cells were used in a co-culture with CD4+ naïve T cells (Lower panel). (B) A representative HPLC profile of 6 independent experiments showing the hydrolysis of exogenous AMP into Adenosine in a co-culture of Treg/CD39+ and CD4+ naïve T cells (Top panel). Addition of the inhibitor of CD73 enzymatic activity inhibits the production of Adenosine in a specific manner (Middle panels). (C) Histograms represent the production of Adenosine form AMP in the co-culture system. (pooled data of 6 independent experiments * P<0.05).
(TIF)

Figure S4 **The capacity of CD39 mAb to inhibit the CD39 ATPase activity.** YT2C2 NK line cells which express high levels of extracellular CD39 were pre-incubated with anti-CD39 mAb (A1) or control IgG1 (10 µg/mL) for 2 h. The cells were then washed with a phosphate-free reaction buffer and ATPase activity was initiated by the addition of ATP at a concentration 100 µM in 200 µl of a phosphate free reaction buffer for 15 min at 37°C. The impact of anti-CD39 mAb was evaluated using HPLC technique using an Ultimate 3000 Thermofisher HPLC coupled with a UV detector on a reverse-phase column (Lichrospher 100-5 RP18 Macherey-Nagel) (A representative Figure of 2 independent experiments).
(TIF)
