# Supplementary figures and images for: Regulatory T Cells Negatively Affect IL-2 Production of Effector T Cells through CD39/Adenosine Pathway in HIV Infection
Source: PLoS Pathog. 2013 Apr 25;9(4):e1003319. doi: 10.1371/journal.ppat.1003319 (PMC3635970; doi:10.1371/journal.ppat.1003319)

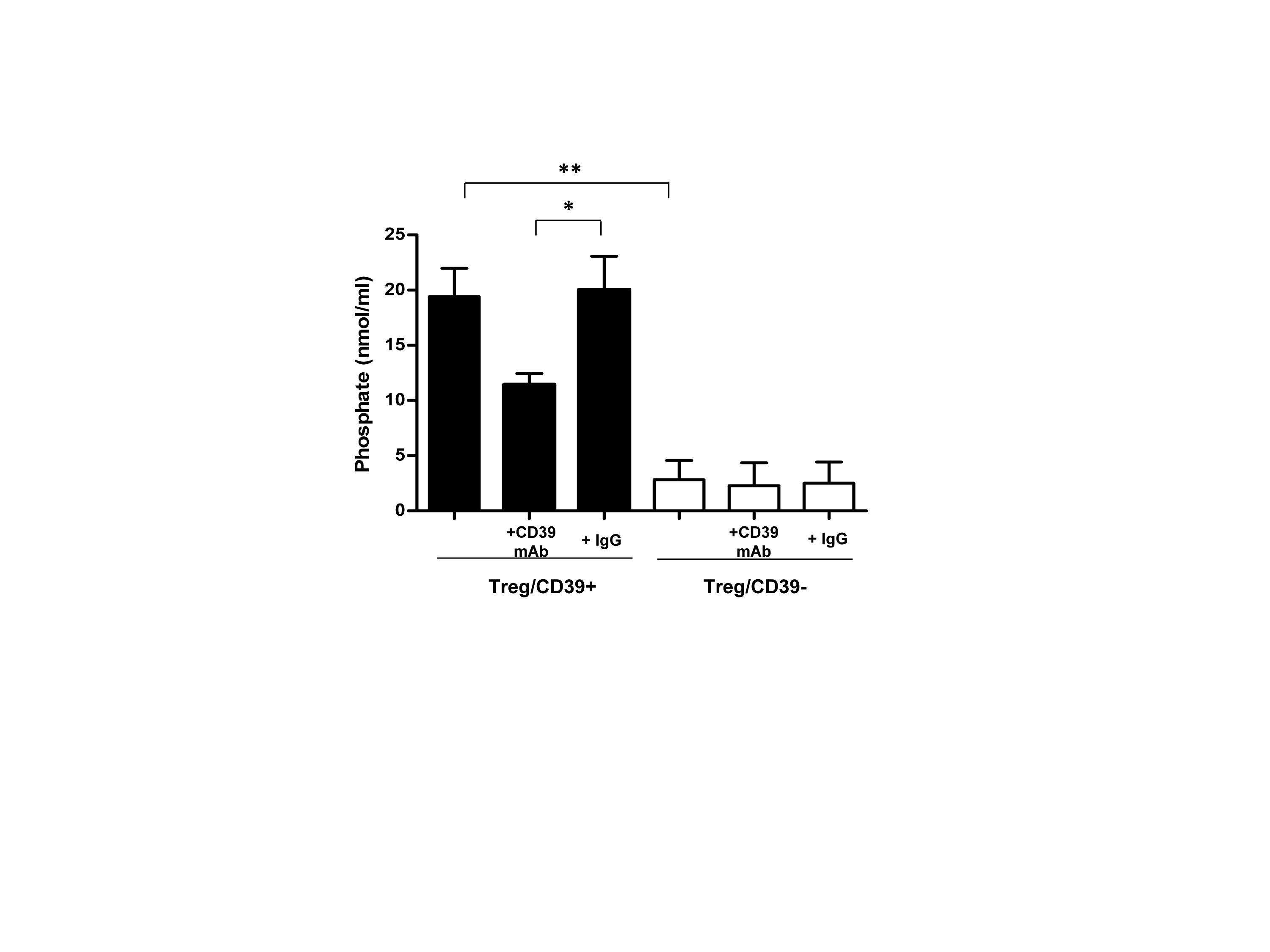

Supplement: Figure S1 — Differential ATPase activity of Treg/CD39+ or Treg/CD39−. FACS-sorted Treg/CD39+ or Treg/CD39− cells were co-cultured with effector CD4+ T cells in the presence or absence of anti-CD39 mAb or control IgG1 (10 µg/mL) for 2 h. The cells were then washed with a phosphate-free reaction buffer and ATPase activity was initiated by the addition of ATP at a concentration 100 µM in 200 µl of the phosphate free reaction buffer for 15 min at 37°C. The released inorganic phosphate by hydrolysis of ATP was measured using the malachite green phosphate detection kit (R&D System, Minneapolis, USA) according to the manufacturer's instructions. Histograms represent Treg/CD39+ capacity to hydrolyse ATP comparing to Treg/CD39−. CD39 mAb inhibits the ATPase activity of CD39 in a specific manner (pooled data of 4 independent experiments, * P<0.05). (TIF) [file ppat.1003319.s001.tif]

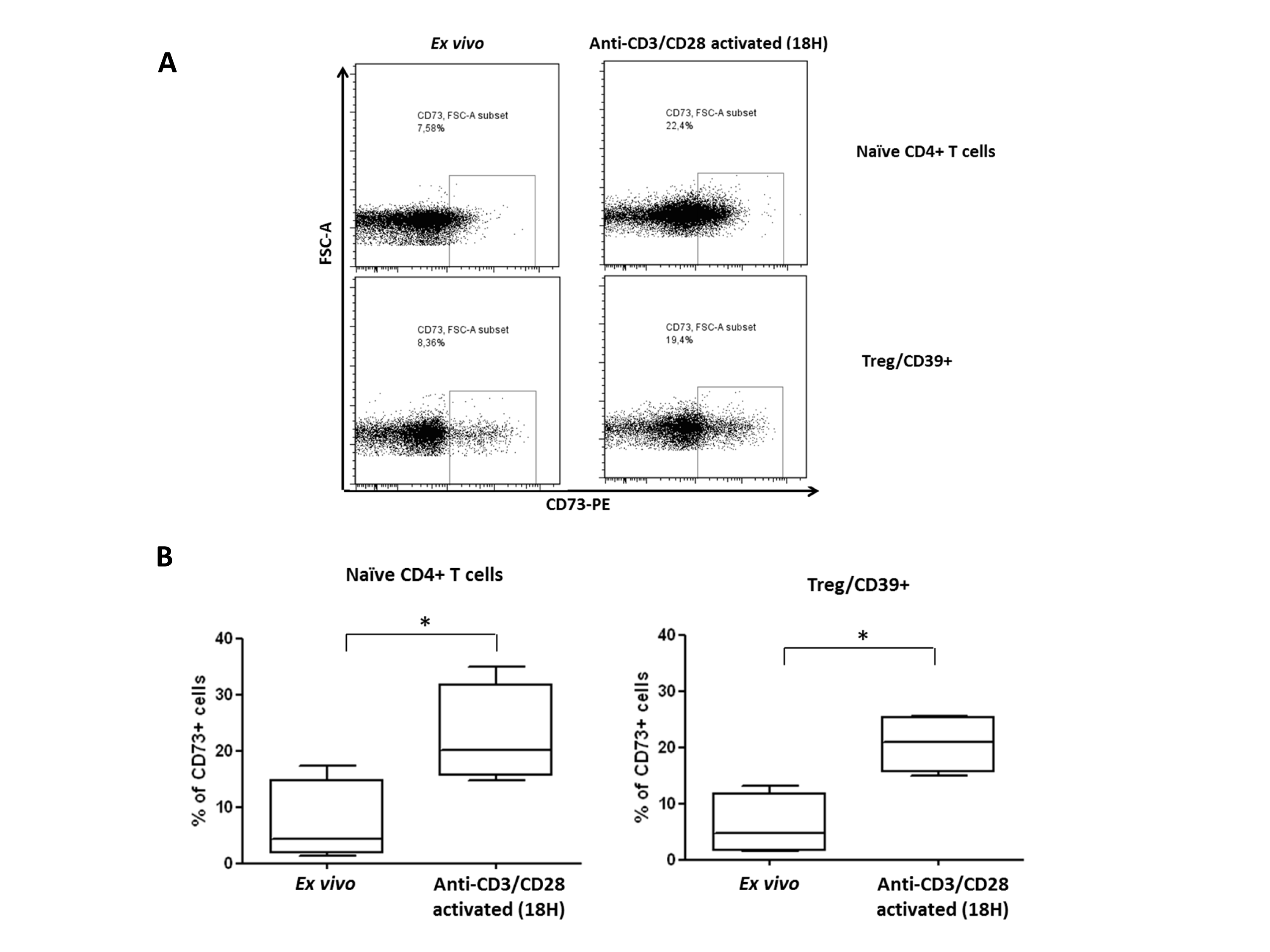

Supplement: Figure S2 — Increase of CD73 expression following overnight anti-CD3/28 mAbs stimulation. FACS-sorted naive CD4+ T cells and Treg/CD39+ cells were activated separately by anti-CD3 and anti-CD28 mAbs (1 µg/mL). After 18H of activation, the cells were washed and stained by anti-CD73 mAb. (A) A representative figure of 5 independent experiments showing an increase of CD73 expression upon anti-CD3/CD28 mAbs stimulation. (B) Histograms represent the increase of CD73 expression upon overnight anti-CD3/CD28 mAbs stimulation. (pooled data of 5 independent experiments * P<0.05). (TIF) [file ppat.1003319.s002.tif]

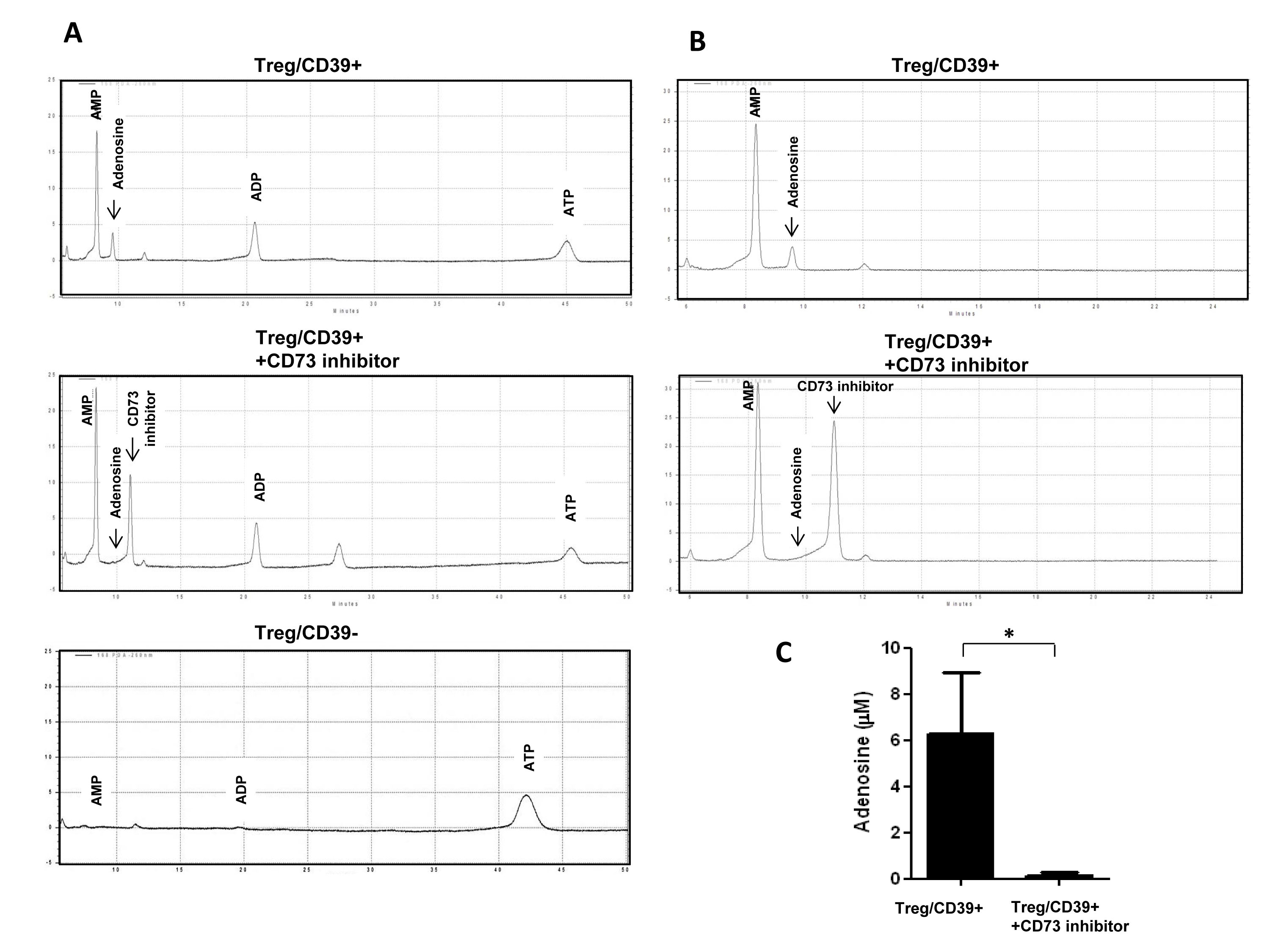

Supplement: Figure S3 — Hydrolysis of ATP or AMP into Adenosine in a co-culture of Treg/CD39+ and naïve CD4 T cells. FACS-sorted Treg/CD39+ or Treg/CD39− cells were co-cultured with anti-CD3/28 mAbs stimulated naïve CD4+ T cells in the presence of 10 µM Dipyridamole to block the transport of Adenosine inside T cells prior to addition of 100 µM ATP or AMP, in 200 µl of RPMI. The cells were incubated for 120 min. with ATP or 45 min with AMP at 37°C, then the hydrolysis of exogenous ATP measured by HPLC. (A) A representative HPLC profile of 4 independent experiments (using a Beckman Coulter System Gold HPLC and a Phenomenex Luna 3u C18 (2) 100A, 150 mm×4.6 mm column) showing the ability of Treg/CD39+ to convert ATP into adenosine (Top panel). Addition of the inhibitor of CD73 enzymatic activity (adenosine 5′-(α, β-methylene diphosphate) inhibits the production of Adenosine in a specific manner (Middle panel). No hydrolysis of exogenous AMP into Adenosine was observed when Treg/CD39− cells were used in a co-culture with CD4+ naïve T cells (Lower panel). (B) A representative HPLC profile of 6 independent experiments showing the hydrolysis of exogenous AMP into Adenosine in a co-culture of Treg/CD39+ and CD4+ naïve T cells (Top panel). Addition of the inhibitor of CD73 enzymatic activity inhibits the production of Adenosine in a specific manner (Middle panels). (C) Histograms represent the production of Adenosine form AMP in the co-culture system. (pooled data of 6 independent experiments * P<0.05). (TIF) [file ppat.1003319.s003.tif]

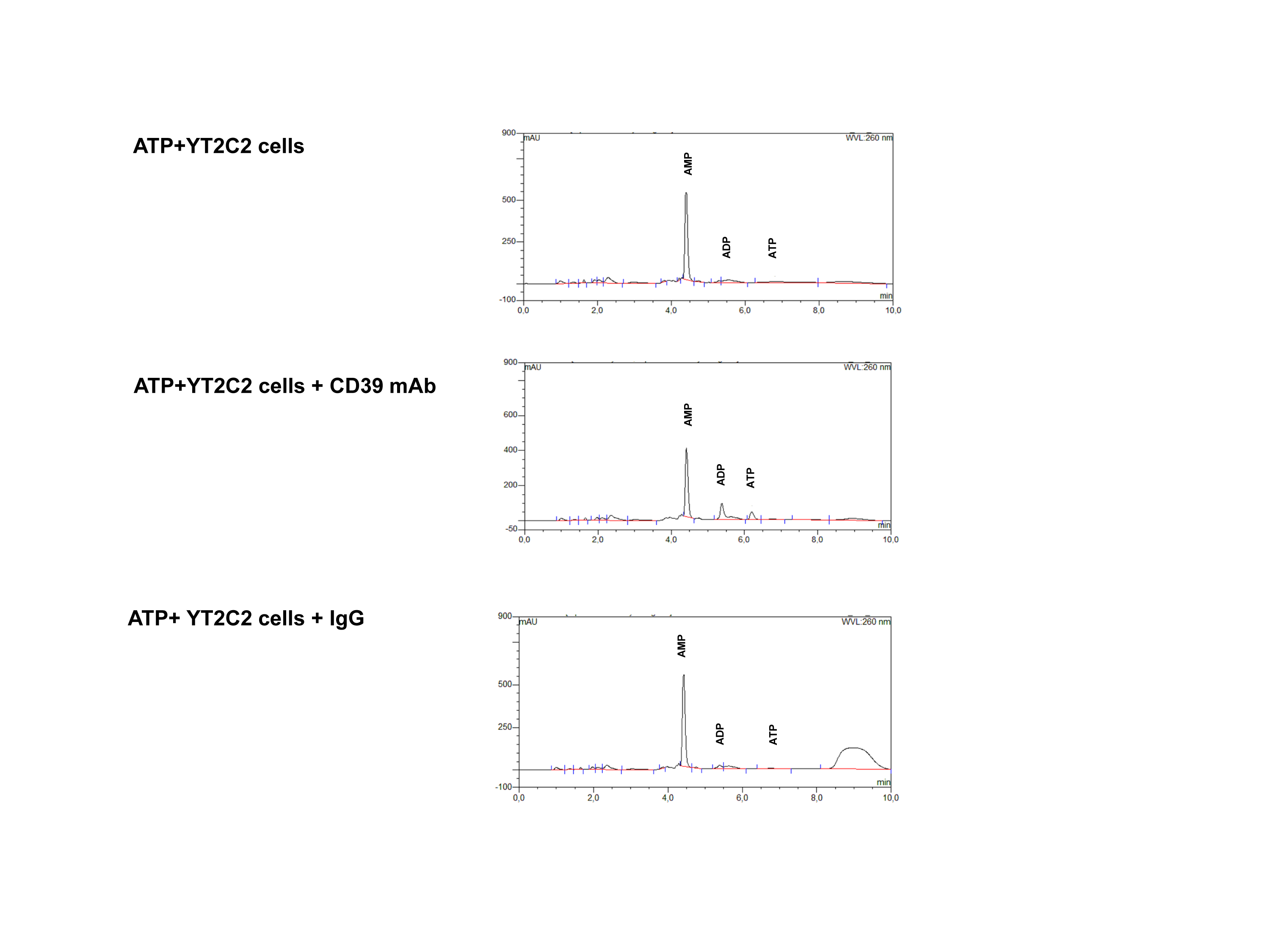

Supplement: Figure S4 — The capacity of CD39 mAb to inhibit the CD39 ATPase activity. YT2C2 NK line cells which express high levels of extracellular CD39 were pre-incubated with anti-CD39 mAb (A1) or control IgG1 (10 µg/mL) for 2 h. The cells were then washed with a phosphate-free reaction buffer and ATPase activity was initiated by the addition of ATP at a concentration 100 µM in 200 µl of a phosphate free reaction buffer for 15 min at 37°C. The impact of anti-CD39 mAb was evaluated using HPLC technique using an Ultimate 3000 Thermofisher HPLC coupled with a UV detector on a reverse-phase column (Lichrospher 100-5 RP18 Macherey-Nagel) (A representative Figure of 2 independent experiments). (TIF) [file ppat.1003319.s004.tif]
